# Enhanced Learning and Memory in Patients with *CRB1* Retinopathy

**DOI:** 10.3390/genes15060660

**Published:** 2024-05-22

**Authors:** Genevieve A. Wright, Ana Catalina Rodriguez-Martinez, Hanne Conn, Mar Matarin, Pamela Thompson, Anthony T. Moore, Rola Ba-Abbad, Andrew R. Webster, Mariya Moosajee

**Affiliations:** 1 Moorfields Eye Hospital NHS Foundation Trust, London EC1V 2PD, UK; genevieve.wright@ucl.ac.uk (G.A.W.); ana.rodriguezmartinez@nhs.net (A.C.R.-M.); tony.moore@ucsf.edu (A.T.M.); rolabaabbad@yahoo.com (R.B.-A.); andrew.webster@ucl.ac.uk (A.R.W.); 2 Institute of Ophthalmology, University College London (UCL), London EC1V 9EL, UK; 3 Department of Clinical and Experimental Epilepsy, Institute of Neurology, University College London Hospitals (UCLH), National Hospital for Neurology and Neurosurgery, Queen Square, London WC1N 3BG, UK; h.conn@ucl.ac.uk (H.C.); m.matarin@ucl.ac.uk (M.M.); pamela.thompson@uclh.nhs.uk (P.T.)

**Keywords:** *CRB1*, inherited retinal diseases, *CRB1* retinopathy, cognitive function, blindness

## Abstract

Mutations in the *CRB1* gene are associated with a diverse spectrum of retinopathies with phenotypic variability causing severe visual impairment. The *CRB1* gene has a role in retinal development and is expressed in the cerebral cortex and hippocampus, but its role in cognition has not been described before. This study compares cognitive function in *CRB1* retinopathy individuals with subjects with other retinopathies and the normal population. Methods: Neuropsychological tests of cognitive function were used to test individuals with *CRB1* and non-*CRB1* retinopathies and compare results with a standardised normative dataset. Results: *CRB1* retinopathy subjects significantly outperformed those with non-*CRB1* retinopathy in list learning tasks of immediate (*p* = 0.001) and delayed memory (*p* = 0.007), tests of *semantic* verbal fluency (*p* = 0.017), verbal IQ digit span subtest (*p* = 0.037), and estimation test of higher execution function (*p* = 0.020) but not in the remaining tests of cognitive function (*p* > 0.05). *CRB1* retinopathy subjects scored significantly higher than the normal population in all areas of memory testing (*p* < 0.05) and overall verbal IQ tests (*p* = 0.0012). Non-*CRB1* retinopathy subjects scored significantly higher than the normal population in story recall, verbal fluency, and overall verbal IQ tests (*p* = 0.0016). Conclusions: Subjects with *CRB1* retinopathy may have enhanced cognitive function in areas of memory and learning. Further work is required to understand the role of *CRB1* in cognition.

## 1. Introduction

Biallelic pathogenic variants in the crumbs cell polarity complex component 1 gene (*CRB1*, OMIM 604210) are associated with a diverse spectrum of retinopathies with phenotypic variability [1]. The phenotypes reported are Leber congenital amaurosis (OMIM #613935, LCA8), early onset severe retinal dystrophy (EOSRD) [2], autosomal recessive retinitis pigmentosa (OMIM #600105, RP12), cone-rod dystrophy (CORD), and macular dystrophy (MD). Distinctive features of *CRB1* retinopathies are nummular pigmentation, fine yellow punctate deposits, preserved para-arteriolar retinal pigment epithelium (PPRPE), and coarse and thickened retina [3,4]. The *CRB1* gene encodes a type 1 transmembrane protein, which is localised to the sub-apical region of the Muller cells and photoreceptors [5]. In the retina, it is known that *CRB1* has a role in retinal development and maintaining retinal integrity, a key component of the zonula adherens junctions at the external limiting membrane (ELM) and contributing to vascular integrity and apicobasal polarity [5]. Optical coherence tomography (OCT) has revealed thicker retinas with abnormal retinal lamination [6,7]. This is attributed to an enriched retinal progenitor population with higher cell proliferation with inhibited cell fate progression, resulting in cell detachment during retinal development [5].

*CRB1* exhibits distinct expression patterns outside the eye. In murine embryos, it is expressed at the ventral area of the neural tube. In adult mouse brains, it is expressed in the cerebellum, hippocampal dentate gyrus, olfactory bulbs, rostral migratory stream, and the subventricular area lining the telencephalic ventricles [8]. Notably, these are the brain areas where neurogenesis occurs [9]. Furthermore, *CRB1* is expressed in other brain areas, including the cerebral cortex and hippocampus (http://proteinatlas.org, accessed on 8 August 2019). The hippocampus is known to be critical for memory and learning, and *CRB1* expression peaks in this region during embryogenesis and early foetal development (http://hbatlas.org/, accessed on 8 August 2019) [10,11,12].

Genetic contribution to human cognition has been established. Within the CA1 region of the hippocampus, the high expression of the *SSRT4*, *PNMT*, *LRTM2*, and *DRD3* genes is critical for memory formation and learning [13]. Disruption of *MDK*, a gene highly expressed in the basal layer of the cerebral cortex with a critical role in neurogenesis [14] and hippocampal development [15], is associated with memory impairment [15]. Pathogenic variants in *BBS1* [16], *CWC27* [17], and *SCAPER* [18] cause a retinal dystrophy with cognitive impairment. However, our understanding of genetic variations that augment cognitive capacities remains limited. Using an established paradigm to study cognitive function, one study reported a family with autosomal dominant cone-rod dystrophy and enhanced cognitive ability, who harboured a heterozygous missense variant (c.2459G > A, p.Arg820His) in *RIMS1*, a gene known to be expressed in the ventricular zone, thalami, and hippocampus areas of the brain [19]. However, they subsequently reported co-inheritance of a heterozygous missense variant (c.1118C > T, p.Arg373Cys) in *PROM1* [20], which is a gene known to be putative in adult hippocampal neurogenesis [21]. It is not known which gene/variant, or if both, may be associated with their augmented cognition. Two kindreds (A and B) with cone-rod dystrophy who harboured the *PROM1* c.1117C > T p.Arg373Cys variant underwent cognitive function testing using the same established paradigm; three out of four affected family members from kindred A displayed average-to-superior verbal memory recall. However, in kindred B, verbal learning was delayed in one affected individual and average in the other [22]. Genes such as *PAX6* are expressed in the eye and brain, but individuals with haploinsufficiency (resulting in aniridia) do not demonstrate enhanced intellectual ability or verbal memory compared with normal controls [23]. Moreover, RasGRF1-deficient mice showed impaired learning abilities and memory performance with retinal degeneration, suggesting an association between retinopathy and memory defects [24]. This study aims to determine whether individuals with *CRB1* retinopathy have enhanced cognitive function in areas of learning and memory, when compared to those with inherited retinal diseases (IRDs) associated with variants in other genes with a comparable degree and onset of visual loss, as well as unaffected individuals from the normal population.

## 2. Materials and Methods

A prospective neuropsychological study from patients with IRDs was performed. Subjects reviewed at a single tertiary referral centre (Moorfields Eye Hospital) with molecularly confirmed *CRB1*-related retinopathy were consecutively recruited, and subjects with molecularly confirmed non-*CRB1* retinopathy and similar level of visual impairment were recruited in parallel. Ethical approval was provided by the Joint Research Ethics Committee of the Institute of Neurology and National Hospital for Neurology and Neurosurgery, Queens Square, and the study adhered to the tenets of the Declaration of Helsinki.

Subjects with a history of other co-morbidities which may impair cognitive function including but not limited to stroke; neurodegenerative disease, e.g., parkinsonism and Alzheimer’s; traumatic brain injury/traumatic amnesia; psychiatric disorders, e.g., schizophrenia and depression; or patients with variants in syndromic retinal genes known to negatively affect cognitive function [e.g., Bardet Biedl syndrome (BBS1)] were excluded.

To account for visual impairment, cognitive function was assessed using standardised neuropsychological tests that did not involve visual processing and required only verbal interaction. The testing protocol was the same as that used for previous collaborative investigations [19,23] and is detailed in the Supplementary Material (see Supplemental Material Table S1). All testing was carried out by a Clinical Neuropsychologist (PT, MM, and HC).

### Statistical Analysis

A two-tailed Mann–Whitney U test was performed to compare mean test scores of *CRB1* retinopathy subjects with non-*CRB1* retinopathy subjects. Mean test scores for both groups were also compared to published normative mean scores (for a standardisation sample (n = 1580) of normally sighted individuals without ocular disease, aged 16–75 years who had undergone neurophysiological testing using the same procedure, see [25,26]), as per previous collaborative investigations [19,23]. Sum of scaled normative data scores SPSS (Version 25) was used to analyse these data.

## 3. Results

### 3.1. Participants

A total of 63 subjects were recruited, 21 with *CRB1* retinopathy and 42 with vision loss due to other inherited ocular conditions. All 63 subjects had a diagnosis of IRDs or congenital visual loss confirmed by clinical examination, visual acuity tests, fundus imaging, and molecular confirmation of pathogenic variants in *CRB1*, *KCNV2*, *BEST1*, *PAX6*, and *RDH12* in accredited clinical laboratories. Four sib-ships and four kindreds were recruited: one sib-ship with *CRB1* and one with *RDH12* retinopathy, two sib-ships and three kindreds with *PAX6*, and one kindred with *PROM1*. As cognitive function is correlated in first-degree relatives, a mean of the scores for each sib-ship and kindred was used in this analysis. The mean age at testing for *CRB1* and non-*CRB1* retinopathy subjects was 36 (SD ± 11.2) and 38 (SD ± 13.1) years, respectively. The mean age of onset of vision loss was 4.1 (SD ± 4.1) and 6.7 (SD ± 4.7) years, respectively. The mean visual acuity at the time of testing was 1.84 (SD ± 0.7) logMAR for *CRB1* retinopathy individuals and 1.4 (SD ± 0.6) logMAR for non-*CRB1* retinopathy individuals. Details of the demographic characteristics of this cohort are reported in Supplementary Table S2.

**Figure 1:**
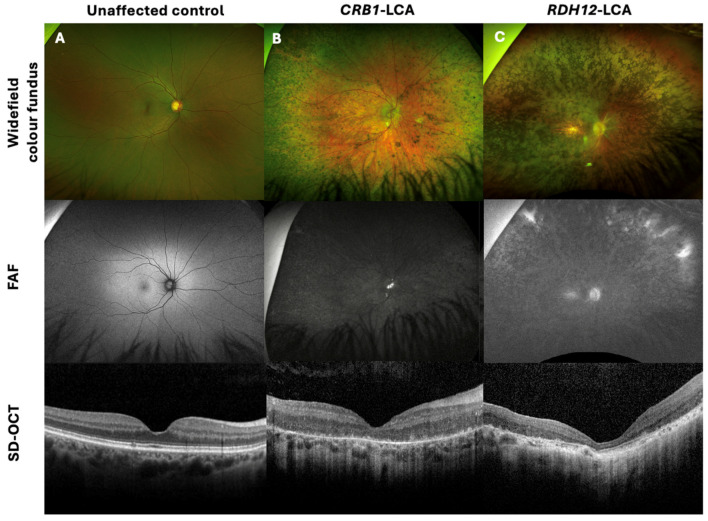
Retinal imaging with widefield colour fundus photography, with corresponding fundus autofluorescence (FAF) and spectral-domain optical coherence tomography (SD-OCT) of a CRB1-Leber congenital amaurosis (LCA) patient, non-CRB1 LCA, and unaffected control. (**A**) Thirty-six-year-old female healthy control with 0.00 logMAR vision in each eye, with normal FAF and SD-OCT imaging. (**B**) Twenty-two-year-old male patient with CRB1-LCA with 1.40 logMAR vision in each eye, displaying widespread nummular pigmentation, dense hypo-AF, and a coarse, thickened retina with retinal disorganisation, with thinning of the outer nuclear layer (ONL) and loss of the ellipsoid zone on SD-OCT imaging. (**C**) Twenty-eight-year-old female patient with RDH12-LCA, presenting with 1.5 logMAR vision in each eye, displaying bone spicules and macular atrophy, dense hypo-AF, and retinal thinning, including the ONL and loss of the ellipsoid zone on SD-OCT.

Common clinical features of *CRB1* retinopathy, as shown in Figure 1A, included widespread nummular pigmentation, peripheral pigment migration, dense hypo-autofluorescence depicted on fundus autofluorescence (FAF) imaging, a coarse, thickened retina with retinal disorganisation, thinning of the outer nuclear layer (ONL), and loss of the ellipsoid zone seen on spectral-domain optical coherence tomography (SD-OCT) imaging. Figure 1C depicts the clinical features of a non-*CRB1* patient with an *RDH12* gene mutation, showing bone spicules, macular atrophy, dense hypo-autofluorescence, and retinal thinning, including thinning of the ONL and loss of the ellipsoid zone on SD-OCT.

#### Subjects with *CRB1* Retinopathy Have Enhanced Cognitive Function

**Figure 2:**
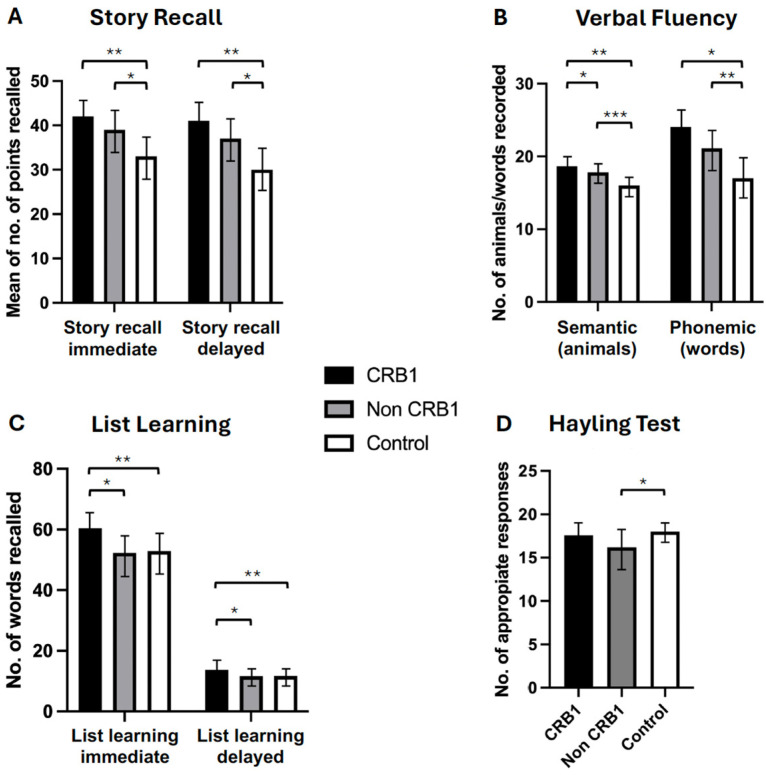
(**A**). Patients with *CRB1* retinopathy and non-*CRB1* retinopathy exhibited no significant differences in immediate and delayed story recall (*p* = 0.111 and *p* = 0.057, respectively). However, both groups displayed significantly higher mean (±SD) scores compared to normal controls in both tasks (* *p* = 0.0001, ** *p* = 0.0001). (**B**). *CRB1* retinopathy subjects scored significantly higher than non-*CRB1* retinopathy patients (* *p* = 0.007), and both *CRB1* retinopathy and non-CRB1 retinopathy subject mean scores (±SD) were significantly higher than normal controls in the semantic (animal) test (** *p* = 0.0001, *** *p* = 0.0001). No significant differences were observed between *CRB1* retinopathy and non-*CRB1* retinopathy subjects in the phonemic fluency tests (*p* = 0.363). However, both *CRB1* retinopathy and non-*CRB1* retinopathy subject mean scores (±SD) were significantly higher than normal controls (* *p* = 0.0001, ** *p* = 0.0001). (**C**). *CRB1* retinopathy patients exhibited significantly higher mean (±SD) scores than non-*CRB1* retinopathy patients in both the list learning immediate and delayed memory tasks (* *p* = 0.001 and * *p* = 0.007, respectively) and normal controls (** *p* = 0.0001 and ** *p* = 0.0004, respectively). No significant differences were observed between non-*CRB1* retinopathy subjects and normal controls in both tasks (*p* = 0.6603 and *p* = 0.8007, respectively) (**D**). No significant differences were found between *CRB1* retinopathy and non-*CRB1* retinopathy subjects (*p* = 0.068) and between *CRB1* retinopathy subjects and normal controls in the Hayling test (*p* = 0.3491). Non-*CRB1* retinopathy subjects scored significantly lower than the normal population (* *p* = 0.0001).

**Figure 3:**
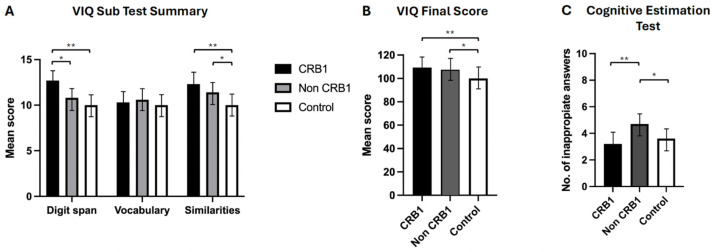
(**A**). *CRB1* retinopathy subjects’ scores were significantly higher compared to non-*CRB1* retinopathy subjects in digit span (* *p* = 0.037), and they outperformed normal controls in digit span (** *p* = 0.0003) and similarities (** *p* = 0.002) but not in vocabulary (*p* = 0.648). Non-*CRB1* retinopathy subjects outperformed normal controls in similarities (* *p* = 0.001) but not in vocabulary (*p* = 0.3666) or digit span (*p* = 0.0600) tests. (**B**). Overall VIQ summary scores showed no significant differences between *CRB1* and non-*CRB1* subjects (*p* = 0.142). Both *CRB1* (** *p* = 0.001) and non-*CRB1* retinopathy (* *p* = 0.001) subjects had significantly higher VIQ scores than the normal population. (**C**). *CRB1* retinopathy subjects had significantly lower scores (indicating greater cognitive ability) than non-*CRB1* retinopathy patients in the cognitive estimation test (** *p* = 0.020), while non-*CRB1* retinopathy subjects scored significantly higher (indicating lower cognitive ability) than normal controls (* *p* = 0.004).

- No significant differences were observed between the two groups of retinopathy subjects in the story recall immediate (*p* = 0.111) and delayed (*p* = 0.057) memory tests [Figure 2A] or in the verbal fluency phonemic subtest (*p* = 0.363) [Figure 2B]. *CRB1* retinopathy subjects scored significantly higher than non-*CRB1* retinopathy subjects in the list learning tasks of immediate (*p* = 0.001) and delayed memory (*p* = 0.007) [Figure 2C], in the verbal fluency semantic subtest (*p* = 0.017) [Figure 2B], and in the Hayling test of mental processing speed (*p* = 0.068) [Figure 2D]. Additionally, *CRB1* retinopathy subjects scored higher in the cognitive estimation test of higher executive function (*p* = 0.020) [Figure 3C] and in the verbal IQ digit span subtest compared to the non-*CRB1* group (*p* = 0.037) [Figure 3A]. No significant differences were found in overall verbal IQ (*p* = 0.142) [Figure 3B] or in the verbal IQ vocabulary (*p* = 0.436) and similarities (*p* = 0.208) subtests [Figure 3A].
- *CRB1* retinopathy subjects scored significantly higher than the normal population in both story recall (*p* = 0.0001) memory tests [Figure 2A], in immediate (*p* = 0.0001) and delayed (*p* = 0.0004) list learning tests [Figure 2C], both verbal fluency tests (*p* = 0.0001) [Figure 3B], in the digit span (*p* = 0.0003) verbal IQ subtest which assesses immediate short term memory recall, verbal IQ similarities subtest (*p* = 0.002), and overall verbal IQ tests (*p* = 0.001) [Figure 3A,B]. There were no significant differences in the Hayling test of mental processing speed (*p* = 0.349) [Figure 2D], in the vocabulary verbal IQ subtest (*p* = 0.648) [Figure 3A], and in cognitive estimation tests of higher executive function (*p* = 0.403) [Figure 3C].
- Non-*CRB1* retinopathy subjects scored significantly higher than the normal population in story recall (*p* = 0.0001) [Figure 2A]. No significant differences were seen in the list learning immediate (*p* = 0.6603) and delayed (*p* = 0.800) memory tests [Figure 2C] and in the verbal IQ digit span (*p* = 0.060) and vocabulary (*p* = 0.366) subtests [Figure 3A]. Additionally, non-*CRB1* retinopathy subjects scored significantly higher than the normal population in both verbal fluency tests (*p* = 0.0001) [Figure 3B], in the similarities verbal IQ subtest (*p* = 0.001) [Figure 3A], and in the overall verbal IQ tests (*p* = 0.001) [Figure 3B]. They, however, scored significantly worse than the normal population in the Hayling test of mental processing speed (*p* = 0.0001) [Figure 2D] and cognitive estimation tests of higher executive function (*p* = 0.004) [Figure 3C].

## 4. Discussion

This is the first study to report elements of enhanced cognitive function in areas of learning and memory in subjects with biallelic *CRB1* retinopathy. *CRB1* patients outperformed non-*CRB1* patients in tests of immediate and delayed memory, higher executive cognitive function, and some aspects of verbal fluency which facilitates stored memory retrieval [27]. They also scored significantly higher than the normal population in all memory and verbal fluency tests, in overall verbal IQ tests, including the digit span subtest which assesses short term memory. Non-*CRB1* retinopathy subjects scored significantly higher than the normal population in verbal fluency tasks and in some, but not all, memory tests, and they scored significantly lower in tests of higher executive cognitive function and mental processing speed.

Individuals with sight impairments are considered to have enhanced working memory capacity [28,29], memory processing [30], and verbal ability [31]. This raises questions about whether there is a causal relationship between variants in *CRB1* and enhanced memory and learning abilities in *CRB1* retinopathy subjects, or if these improvements are the consequence of early-onset blindness, a known factor that triggers compensatory neuroplasticity and the reorganisation of neural circuits [32,33]. Careful control for the effects of blindness in different groups was undertaken, by comparing the *CRB1* retinopathy group to a group of subjects with a similar age of onset of visual impairment and similar degree of sight impairment. The mean age of onset of vision loss was 4.1 (SD ± 4.1) in *CRB1* patients and 6.7 (SD ± 4.7) years in non-*CRB1* retinopathy, and the mean visual acuity was 1.84 (SD ± 0.7) logMAR and 1.4 (SD ± 0.6) logMAR, respectively. Additionally, careful control for the known effects of increased age on cognitive function [34] was undertaken by having groups with similar mean ages 36 (SD ± 11.2) years for *CRB1* patients and 38 (SD ± 13.1) years for non-*CRB1* retinopathy patients). The findings of this study suggest that biallelic *CRB1* pathogenic variants could further enhance cognitive function in subjects with visual impairment.

Previous evidence suggests an association between genetic mutations in eye–brain-expressed genes and the enhancement of some areas of cognitive function in humans. A kindred of eight individuals with variants in *PROM1* c.1117C > T p.Arg373Cys and *RIMS1* c.2459G > A, p.Arg820His and seven unaffected individuals underwent cognitive testing. Affected individuals were found to have above average scores in verbal IQ tests that involved nonvisual processing. However, their scores in memory and executive function tests were variable, with some scores below average. The enhancement of some cognitive domains was attributed to genetic mutation rather than impaired visual function [19,20]. A kindred of four with the same *PROM1* c.1117C > T p.Arg373Cys variant displayed superior memory recall. However, one individual was found to have delayed learning [22]. Wang et al. observed the enhancement of recognition memory in the murine forebrain following the overexpression of type-1 adenylyl cyclase (*ADCY1*), a gene known to be crucial in hippocampus memory formation by increasing cyclic AMP, which positively regulates synaptic plasticity. They postulated that a shift in the balance between negative and positive regulators whose activities counteract one another, e.g., protein phosphatases versus protein kinases, could occur following transgenic overexpression, may induce further synaptic plasticity, and that the enhancement of protein kinase (MAPK) signalling can increase memory recognition. However, they also suggested that such an enhancement of one area of memory had the potential to impair the function of another [35].

Earlier studies have shown that mutations in the CA1 hippocampal region-expressed genes have detrimental effects on hippocampus-dependent memory tasks: in *CNB1* gene knockout mice, working memory and synaptic plasticity were impaired, suggesting that rather than enhancing performance, the mutation induces the inhibition of crucial components of memory regulation [36]. Moreover, deletion of the ryanodine receptor type 3 (*RyR3*) gene in mice has been shown to impair some forms of hippocampal synaptic plasticity and spatial learning. *RyR3* deletion negatively affects long-term potentiation and depotentiation in the hippocampal CA1 region and dentate gyrus, leading to deficits in hippocampal synaptic plasticity and reduced performance in spatial memory tasks [37]. No memory impairment was observed in our *CRB1* retinopathy subjects; in fact, all areas of memory not reliant on vision were enhanced compared to the normal population.

Molecular changes in brain-expressed genes have been found to positively influence memory and other areas of cognition [38]. Deletion of 5-HT1B (a gene thought to modify cognitive behaviour) in mice resulted in enhanced spatial memory performance and facilitated learning abilities [39]. The NR2B (N-methyl-D-aspartate) receptor, a synaptic coincidence detector critical for learning and memory formation, acts as a “molecular switch” in the memory process. Tang et al. demonstrated that *NR2B* transgene overexpression in the cortex and hippocampus of adult mice resulted in superior performance in several hippocampus-dependent learning and memory tasks [40,41] and they outperformed age-matched controls into old age [42]. Enhanced cognition is also associated with variants in further brain-expressed genes cb1-b [43], TLCN [44], and CREB [45].

*CRB1* is expressed in the hippocampus, an area of the brain critical for learning and memory [46]. However, the association of *CRB1* with memory and learning is yet to be explained. Biallelic *CRB1* mutation causes isolated retinal dystrophy without brain dysfunction. The overexpression of CRBE2 (a member of the crumbs cell polarity complex, expressed in retinal pigment epithelium and the cortex, hippocampus, hypothalamus, and cerebellum of adult mouse brains, and an essential regulator for neuronal differentiation during neurogenesis) is induced in the cortex of the CRB1rd8 mutant mouse brain, suggesting a possible compensatory mechanism for CRB1 dysfunction [47]. CRB1 is integral to tight junctions and apical-basal polarity and the lack of it results in increased cell proliferation in the retina and cells in a proliferative phase [5]. If this was also seen in the hippocampus, maybe there is an increased number of cells and loss of lamination that may allow for increased neuronal growth/synaptic plasticity. This expression occurs in foetal stages, so increased cell proliferation in that area may enhance memory capacity. However, further studies are needed to prove this hypothesis.

There is evidence that early-onset blindness can result in training-induced neuroplastic changes in humans [48] and animals, whereby if one cortical area is deprived of adequate sensory input during early development, the reorganisation of the non-deprived senses occurs by means of cross-modal synaptic plasticity [49,50]. Loss of function mutations in the cone-rod homeobox-containing gene (*CRX*) in knockout mice demonstrated sensory compensation and striking adaptions to their environment by utilising non-visual information 5 days after visual loss [51]. Moreover, decades of research in humans suggested that congenital blindness results in superior memory [28,29,30,32,33,52,53] and learning abilities [31] resulting from environmentally induced compensatory alterations within the occipital cortex [54,55]. fMRI studies have demonstrated that sensory reorganisation and recruitment of other sensory modalities occurs in the congenitally blind brain, with increased activation and neuroplastic changes occurring within the occipital cortex [56,57,58]. Whether these striking adaptions and enhancement of memory and learning in the blind brain is entirely environmentally dependent or whether variants in genes also expressed in the brain can increase the potential for synaptic plasticity is yet to be established.

Investigation of whether there is a correlation between the severity of *CRB1* retinopathy and memory enhancement could lead to valuable insights, particularly considering the established link between compensatory cross-modal neuroplasticity in blind individuals and the extent of their visual impairment [28,59]. Furthermore, assessing the cognitive abilities of *CRB1* knockout mice or other associated disease models could offer a different perspective on its function in the brain and whether its absence induces synaptic neuroplasticity.

### Limitations

There are some limitations to our study: the raw normative dataset was not available to the investigators, therefore it was only feasible to perform statistical analysis using mean values, instead of median values, for each group of subjects. It was not possible to control for the known effects of gender, educational level, and socio-economic status on cognitive function [60] as this would have significantly reduced the sample size. A further control group of *CRB1* carriers was not available. It is noteworthy that whilst both retinopathy groups had similar visual acuity levels at time of testing, 68% of *CRB1* retinopathy subjects had unrecordable or severely restricted visual fields, which would have further affected overall levels of visual impairment for this group, whereas only a proportion (28%) of the non-*CRB1* retinopathy subjects had field loss. This could have influenced test performance, given that the enhancement of memory performance appears to be more profound in individuals who have no useful measurable vision [28,59]. A total of 80% of *CRB1* retinopathy subjects had achieved a minimum of A-level education compared with 55% of subjects in the non-*CRB1* retinopathy group. This could imply that the *CRB1* group was biased in relation to educational attainment, particularly as educational level and cognitive function positively correlate [60]. However, it may also suggest that patients with *CRB1* retinopathy have superior learning abilities, which concurs with their significantly higher scores in the list learning and semantic verbal fluency tasks. Finally, *PAX6* mutation patients were included as part of the non-*CRB1* group, *PAX6* mutations play a fundamental role in brain development, and there is evidence that *PAX6* mutations correlate with anatomical and functional changes [61].

## 5. Conclusions

Understanding the molecular processes involved in memory is a challenging and growing field. Any Mendelian disorder, such as *CRB1* retinopathy, in which cognition appears enhanced suggests a biological pathway of importance in this process that is yet to be discovered. This study provides insights into the role of *CRB1* in the eye and brain, and further studies are required to investigate how its dysfunction can lead to enhanced cognition.

## Data Availability

The original contributions presented in the study are included in the article/Supplementary Material, further inquiries can be directed to the corresponding author.

## Supplementary Materials

The following supporting information can be downloaded at https://www.mdpi.com/article/10.3390/genes15060660/s1, Table S1: Summary of neurophysiological testing protocol; Table S2: Summary of subject demographics, genetic results, and clinical characteristics of all 21 patients with biallelic pathogenic variants in *CRB1* and 42 patients with variants in other genes (*BEST1*, *KCNV2*, *PROM1*, *PAX6*, *RDH12*) [25,26,62,63,64,65,66].
