# Supplementary material for: Enhanced Learning and Memory in Patients with CRB1 Retinopathy
Source: Genes (Basel). 2024 May 22;15(6):660. doi: 10.3390/genes15060660 (PMC11203261; doi:10.3390/genes15060660)
Supplement: Supplementary file 1 [file genes-15-00660-s001.zip › untitled folder/Supplementary table S2.docx]

**Supplementary table 2.** Summary of subject demographics, genetic results, and clinical characteristics of all 21 patients with biallelic pathogenic variants in *CRB1* and 42 patients with variants in other genes (*BEST1, KCNV2, PROM1, PAX6, RDH12*)

| **Family number** | **Gene mutation** | **Gender** | **Ethnicity** | **Age** | **Phenotype** | **Zygosity** | **Variant 1 cDNA**  **Variant 1 protein** | **Variant 2 cDNA**  **Variant 2 protein** | **BCVA RE** | **BCVA LE** |
| --- | --- | --- | --- | --- | --- | --- | --- | --- | --- | --- |
| 2630 | *CRB1* | M | Unknown | 35 | LCA/EOSRD | Compound heterozygous | 1576C>T; p.Cys948Tyr | 2843G>A  p.Arg526Xaa | 2.28 | 3 |
| 16291 | *CRB1* | M | White-British | 19 | LCA/EOSRD | Compound heterozygous | c. 2290C>T  p.Arg764Cys | c.2401A>T  p.Lys801Ter | 0.8 | 0.7 |
| 18231 | *CRB1* | F | White-British | 66 | LCA/EOSRD | Compound heterozygous | c.584G>T  p.Cys195Phe | c.3442T>C  p.Cys1148Arg | 1.3 | 1.3 |
| 4564 | *CRB1* | F | Other | 26 | LCA/EOSRD | Compound heterozygous | c.584G>T  p.Cys195Phe | c.2843G>A p.Cys948Tyr | 2.28 | 1.3 |
| 2969 | *CRB1* | F | White-British | 32 | LCA/EOSRD | Compound heterozygous | c.3037C>T  p.Phe1125Leu | c.3914C>T,  p.Pro1305Leu | 2.28 | 1 |
| 17825 | *CRB1* | M | White-British | 31 | LCA/EOSRD | Compound Heterozygous | c.2401A>T p.Lys801Ter | c.3320T>C p.Leu1107Pro | 2.28 | 2.70 |
| 18361 | *CRB1* | M | White British | 43 | LCA/EOSRD | Compound Heterozygous | c.2688T>A  p.Cys896Ter | c.2843G>A p.Cys948Tyr | 2.7 | 2.28 |
| 18047 | *CRB1* | M | Other | 27 | LCA/EOSRD | Compound Heterozygous | c.2843G>A p.Cys948Tyr | c.728_744del  p.Leu243ArgfsTer12 | 1.7 | 1.7 |
| 3164 | *CRB1* | M | Unknown | 54 | LCA/EOSRD | Compound heterozygous | ex6 2025G>T p. | ex9 3520T>G p.C1174G | 0.17 | 1 |
| 16595 | *CRB1* | M | White | 42 | RP | Compound Heterozygous | c.2222T>C  p.Met741Thr | c.2843G>A p.Cys948Tyr | 2.28 | 2.28 |
| 17009** | *CRB1* | M | White | 39 | RP | Homozygous | c.2290C>T  p.Arg764Cys | | 2.28 | 2.70 |
| 17009** | *CRB1* | M | White | 37 | RP | Homozygous | c.2290C>T  p.Arg764Cys | | 2.28 | 1.7 |
| 17009** | *CRB1* | F | White | 42 | RP | Homozygous | c.2290C>T  p.Arg764Cys | | 0.44 | 0.44 |
| 17463 | *CRB1* | M | Unknown | 45 | LCA/EOSRD | Compound Heterozygous | c.2548G>A; p.Pro1381Leu | c.4142C>T p.Gly850Ser | 2.28 | 2.28 |
| 3642 | *CRB1* | M | Unknown | 24 | LCA/EOSRD | Homozygous | c. 750T>G  p.Cys250Trp | | 2.28 | 0.40 |
| 3760 | *CRB1* | M | Asian-Pakistani | 26 | RP | Homozygous | c.2536 G>A  p.Gly846Arg | | 1.3 | 1.00 |
| 16429 | *CRB1* | M | White-British | 21 | LCA/EOSRD | Homozygous | c.2843G>A  p.Cys948Tyr | | 1.3 | 1.3 |
| 3798 | *CRB1* | M | White/Black African | 46 | LCA/EOSRD | Homozygous | c.3074G>A  p.Ser1025Asn | | 2.28 | 2.70 |
| 3671 | *CRB1* | M | Unknown | 42 | LCA/EOSRD | Compound heterozygous | c.2842+5G>A | c.3879-1203C>G | 2.7 | 2.7 |
| 20409 | *CRB1* | M | Unknown | 26 | LCA/EOSRD | Compound Heterozygous | c.584G>T p.Cys195Phe | c.2843G>A p.Cys948Tyr | 2.28 | 2.28 |
| 18025 | *CRB1* | M | Unknown | 41 | LCA/EOSRD | Monoallelic CRB1 | c.584G>T  p.Cys195Phe | | 1.8 | 1.8 |
| 18207 | *KCNV2* | F | Unknown | 51 | CORD | Homozygous | c.1381G>A  p.Gly461Arg | | 1 | 1 |
| 14604 | *KCNV2* | F | White-British | 20 | CORD | Homozygous | c.1316C>T  p.Thr439Ile | | 0.18 | 0.3 |
| 16982 | *KCNV2* | M | White-British | 66 | CORD | Homozygous | c.7A>T  p.Lys3Stop | | 1 | 1 |
| 17589 | *KCNV2* | F | Unknown | 27 | CORD | Homozygous | c.1404del  p.H468fsX503 | | 1 | 1 |
| 4052 | *KCNV2* | F | White-British | 40 | CORD | Compound heterozygous | c.1199del p.Phe400Serfs*54 | c.8_11del p.Lys3Argfs*96 | 0.6 | 0.7 |
| 15870 | *KCNV2* | M | Asian - Pakistani | 31 | CORD | Homozygous | c.916G>T  p.Glu306* | | 0.7 | 0.7 |
| 16962 | *KCNV2* | F | White-British | 40 | CORD | Compound heterozygous | c.433C>T p.Glnl45Ter | c.776C>T  p.Ala259Val | 0.9 | 1.3 |
| 18136 | *KCNV2* | F | Unknown | 41 | CORD | Compound heterozygous | c.1381G>T p.Gly461Ter | c.1638G>T  p.Ter546Tyrext*60 | 1 | 0.9 |
| 13534* | *PAX6* | M | White-British | 32 | Aniridia | Heterozygous | c.1125C>T p.Glu228GlyfsX5 |  | 2.7 | 2.28 |
| 13534* | *PAX6* | M | White-British | 54 | Aniridia | Heterozygous | c.1125C>T p.Glu228GlyfsX5 |  | 1 | 1 |
| 13930 | *PAX6* | F | White-British | 21 | Aniridia | Heterozygous | c.459dup; p.Gly154Argfs*46 |  | 0.6 | 1 |
| 15603** | *PAX6* | M | White-British | 42 | Aniridia | Heterozygous | c.718C>T;  p.Arg240X |  | 1.4 | 1.4 |
| 15593 | *PAX6* | F | White-British | 52 | Aniridia | Heterozygous | c.1601delT fs. |  | 3 | 1 |
| 11553* | *PAX6* | F | White-British | 31 | Aniridia | Heterozygous | c.553G>T p.Gly64Val |  | 1.4 | 1.4 |
| 14039 | *PAX6* | F | Unknown | 38 | Aniridia | Heterozygous | PAX6 IVS6+2insT splicing. |  | 1 | 1 |
| 13482 | *PAX6* | M | Unknown | 35 | Aniridia | Heterozygous | IVS6+2 ins TT. |  | 0.7 | 0.7 |
| 15603** | *PAX6* | F | White-British | 34 | Aniridia | Heterozygous | c.718C>T p.Arg240Ter |  | 3 | 3 |
| 12591 | *PAX6* | F | White-British | 43 | Aniridia | Heterozygous | c.1268A>T  p.Gln423Leu |  | 1 | 1 |
| 12419 | *PAX6* | F | White-British | 34 | Aniridia | Heterozygous | Exon 6 splice mutation-precise details unknown |  | 1.7 | 1.7 |
| 14206 | *PAX6* | F | White-British | 17 | Aniridia | Heterozygous | c.372C>A p.Asn124Lys |  | 3 | 1.3 |
| 10689** | *PAX6* | M | White-British | 35 | Aniridia | Heterozygous | c.1137dupT  p.Ser259 |  | 1.4 | 1.3 |
| 10689** | *PAX6* | M | White-British | 32 | Aniridia | Heterozygous | c.1137dupT  p.Ser259PhefsX2 |  | 1.3 | 1.3 |
| 14428* | *PAX6**** | F | Unknown | 17 | Aniridia | Heterozygous | c.683-6T>A  p.Glu228Glyfs*5 |  | 1.3 | 1.3 |
| 16353 | *PAX6* | M | Other | 37 | Aniridia | Heterozygous | Intron 6 IVS6+5G>A. |  | 1.7 | 1.7 |
| 14428* | *PAX6* | F | White-British | 45 | Aniridia | Heterozygous | c.1253_1262del10  p.Trp418Tyrfs*38 |  | 1.4 | 2.28 |
| 19404 | *PAX6* | M | Unknown | 31 | Aniridia | Heterozygous | c.1080C>T p.Arg240Ter |  | 0.7 | 0.7 |
| 11553* | *PAX6* | F | White-British | 74 | Aniridia | Heterozygous | c.553G>T p.Gly64Val |  | 1 | 1 |
| 17723* | *PROM1* | F | White-British | 44 | *PROM1*-related retinal degeneration | Heterozygous | c.1117C>T  p.Arg373Cys |  | 0.30 | 1.40 |
| 17723* | *PROM1* | F | White-British | 21 | PROM1-related retinal degeneration | Heterozygous | c.1117C>T  p.Arg373Cys |  | 1.6 | 1.8 |
| 17498 | *RDH12* | F | White-British | 18 | LCA/EOSRD | Compound  heterozygous | c.806_810del  p.Ala269GlyfsTer2 | c.144 C>T  P.Asn48= | 1.4 | 2.7 |
| 11968 | *RDH12* | M | Unknown | 38 | LCA/EOSRD | Homozygous | c.146C>A  p.Thr49Lys | | 2.28 | 2.28 |
| 18365 | *RDH12* | F | Asian-Indian | 56 | LCA/EOSRD | Homozygous | c.146C>T  p.Thr49Met | | 2.7 | 2.7 |
| 3169 | *RDH12* | F | White-any other | 47 | LCA/EOSRD | Compound Heterozygous | c.295C>A  p.Leu99= | c.883C>T  p.Arg295Ter | 2.7 | 2.7 |
| 5030 | *RDH12* | M | Other | 30 | LCA/EOSRD | Homozygous | c.609C>A  p.Ser203Arg | | 1.7 | 1.7 |
| 3012 | *RDH12* | F | Unknown | 44 | LCA/EOSRD | Homozygous | c.619A>G  p.N207D | | 1 | 1 |
| 15169** | *RDH12* | M | Asian-Pakistani | 31 | LCA/EOSRD | Homozygous | c.619A>G  p.Asn207Asp | | 2.28 | 2.28 |
| 15169** | *RDH12* | F | Asian-Pakistani | 36 | LCA/EOSRD | Homozygous | c.619A>G  p.Asn207Asp | | 2.7 | 2.7 |
| 18679 | *BEST1* | F | White | 33 | ADBMD | Homozygous | c.901G>A  p.Asp301Asn | | 0.7 | 1 |
| 18448 | *BEST1* | M | Unknown | 43 | ARB | Homozygous | c.468C>G  p.His156Gln | | 1 | 0.7 |
| 4124 | *BEST1* | M | White-British | 69 | ARB | Homozygous | c.974T>C  p.Met325Thr | | 1 | 1.7 |
| 5025 | *BEST1* | F | Unknown | 38 | ARB | Compound heterozygous | c.122C>T; p.Leu41Pro | c.422G>A; p.Arg141His | 0.7 | 1 |
| 17804 | *BEST1* | M | Unknown | 23 | ARB | Homozygous | c.636+1g>c | | 0.6 | 0.7 |
| EOSRD, Early onset severe retinal dystrophy; LCA, Leber congenital amaurosis; RP, Retinitis pigmentosa; MD, Macular dystrophy; CORD, Cone-rod dystrophy; CD, Cone dystrophy; ARK, aniridia related retinopathy; ADBVMD Autosomal dominant best macular dystrophy; ARB autosomal recessive bestrophinopathy; MD Macular dystrophy; BCVA, best corrected visual acuity.  BCVA reported in Logmar.  Counting fingers vision was given a LogMAR value of 1.98, hand motion of 2.28, light perception of 2.7 and no light perception of 3.  *Kindred/parent-child  ** sibship  *** PAX6 mutation inherited paternally | | | | | | | | | | |
