# Supplementary material for: Enhanced Learning and Memory in Patients with CRB1 Retinopathy
Source: Genes (Basel). 2024 May 22;15(6):660. doi: 10.3390/genes15060660 (PMC11203261; doi:10.3390/genes15060660)
Supplement: Supplementary file 1 [file genes-15-00660-s001.zip › untitled folder/Supplemental material S1.docx]

| **Test**  *Subtest* | **Description** |
| --- | --- |
| **Verbal IQ (Wechsler Adult Intelligence tests)** | Measures general intelligence and enables discrimination of verbal reasoning at a higher level of cognitive functioning and strongly correlates with verbal intelligence [1,2] The Verbal IQ test is the only means of assessing the intellectual functioning of visually impaired individuals and has long been considered an appropriate method of assessment [3] |
| *Similarities test* | Measures the subject’s ability to understand a wide-ranging vocabulary. |
| *Digit span* test. | Comprises the Digit Forward and Digit Backward tests and assesses the subjects’ immediate  (short term) auditory memory recall and freedom from distraction. |
| *Vocabulary test* | Measures expressive word knowledge |
| **Verbal Memory test** | This uses tasks derived from the Adult Memory Processing Battery [4]. The tests are designed to measure components of memory, namely retention and learning (only verbal sub tests were used for the purpose of this study). |
| *Story Recall Test* | Assesses immediate and delayed ability to recall a short passage and the ability to immediately register verbal information and retain it over time |
| *List Learning Task* | Assesses learning of verbal information and susceptibility to interference. |
| **Verbal Fluency Test** | Two tests routinely used during neuropsychological assessments [5] were chosen to assess aspects of verbal fluency |
| *Phonemic fluency* | Measures the subject’s ability to recite as many words beginning with the letter S in 60 seconds. |
| *Semantic fluency* | Measures the subjects ability to recite as many animals as they can in 60 seconds. |
| **Mental processing (Hayling Test)** | Assesses mental processing speed *–* This is a response suppression task using the Hayling Test [6] whereby the subject is read two sets of 15 sentences which are all missing the last word. In the first set a sensible completion of each sentence is required and in  the second set a nonsensical completion is required by the subject. |
| **Cognitive Estimation test** | Assesses key areas of problem solving. It quantifies the subject’s ability to retrieve and manipulate particular details from a cognitive set and monitors the appropriateness of their response to simple questions. It has become a widely used test of higher executive functions in the UK [7]. In this test the lower the score the higher the performance. |

**Table S1**. Summary of neurophysiological testing protocol.

**Supplementary references:**

1. Wechsler, D. WAIS-R *Administration and scoring manual*, New York Psychological corporation, 1981

2. Wechsler, D. WAIS-III: *Administration and scoring* ma*nual: Wechsler Adult Intelligence Scale*—*third edition*. San Antonio, TX: The Psychological Corporation, 1997

3.. Price, J. R., Mount, G. G., and Coles, E. A.. *Evaluating the visually impaired: neuropsychological technique*. Journal of Visual Impairment and Blindness, 1987. 48, 20–30.

4. Coughlan, A. K. and Hollows, S. E*. The adult memory and information processing battery (AMIPB) test manual*. Publication A. K. Coughlan, St James University Hospital, Leeds, 1986

5. Spreen,O and Strauss, E. *A compendium of Neuropsychological Tests*. Oxford University Press. New York, 1998.

6. Burgess PW, and Shallice T. *The Hayling Island and Brixton Test manual*. Bury St Edmunds: Thames Valley Test Co., 1997.

7. [Shallice T](http://www.ncbi.nlm.nih.gov/pubmed/?term=Shallice%20T%5BAuthor%5D&cauthor=true&cauthor_uid=679710) and [Evans ME](http://www.ncbi.nlm.nih.gov/pubmed/?term=Evans%20ME%5BAuthor%5D&cauthor=true&cauthor_uid=679710). *The involvement of the frontal lobes in cognitive estimation*. [Cortex.](http://www.ncbi.nlm.nih.gov/pubmed/679710) 1978. Jun;14(2):294-303
